# Fine scale infectious disease modeling using satellite-derived data

**DOI:** 10.1038/s41598-021-86124-2

**Published:** 2021-03-25

**Authors:** Nistara Randhawa, Hugo Mailhot, Duncan Temple Lang, Beatriz Martínez-López, Kirsten Gilardi, Jonna A. K. Mazet

**Affiliations:** 1 One Health Institute, School of Veterinary Medicine, University of California, Davis, USA; 2 University of California, Davis, USA; 3 Department of Statistics, University of California, Davis, USA; 4 Center for Animal Disease Modeling and Surveillance, Department of Medicine and Epidemiology, School of Veterinary Medicine, University of California, Davis, USA

**Keywords:** Computational models, Viral infection

## Abstract

Innovative tools for modeling infectious agents are essential for better understanding disease spread given the inherent complexity of changing and interacting ecological, environmental, and demographic factors. We leveraged fine-scale satellite data on urban areas to build a road-connected geospatial network upon which to model disease spread. This model was tested by simulating the spread of the 2009 pandemic influenza in Rwanda and also used to determine the effects of vaccination regimens on outbreak spread and impact. Our results were comparable to data collected during the actual pandemic in Rwanda, determining the initial places affected after outbreak introduction in Kigali. They also highlighted the effectiveness of preventing outbreaks by targeting mitigation efforts at points of outbreak origin. This modeling approach can be valuable for planning and control purposes in real-time disease situations, providing helpful baseline scenarios during initial phases of outbreaks, and can be applied to other infectious diseases where high population mobility promotes rapid disease propagation.

## Introduction

Anthropogenic activities have transformed one-third to one-half of Earth's land surface, and human-altered landscapes continue to expand^1^. Changing ecological, environmental, and demographic conditions have led to the increased occurrence of emerging infectious diseases (EIDs) that pose an imminent threat to human health and have cost hundreds of billions of US dollars globally in economic losses over the past decades^2,3^. Simultaneously, the unprecedented speed, expansion, and volume of human travel has facilitated the rise of pandemics by coupling infectious disease spread with global transport^4^. Infectious disease dynamics are influenced by inherent stochasticity of real-world phenomena, i.e. effects of chance and non-sequential event progression, consequently making mathematical models and computational tools essential for representing the complexity of these processes, although achievement of complete accuracy remains difficult^5^.

The spatio-temporal progression of outbreaks has commonly been studied using metapopulation models, wherein a population is divided into spatially explicit subpopulations connected to each other by human movements, such as transportation by road or air. One such model, the GLobal Epidemic and Mobility Computational Model (GLEaM), allows the global spatio-temporal characterization of epidemics^6^. The GLEaM model incorporates both air travel and local commuting by partitioning geographic census areas based on their distances to nearby airports and extrapolating commuting processes between them^6^. GLEaM has been found to be accurate and generalizable for determining global disease spread; however, it cannot be applied to determining the regional spread of diseases where few or no airports exist. We build upon GLEaM by changing the underlying network structure and subsequent regional mobility flow for areas inadequately serviced by airports, though they may still be susceptible to EID events. We use high resolution satellite imagery of urban built-up areas^7^ in combination with population and road data to create a regional network of urban areas coupled with underlying population information and connections via roads. The resultant model incorporates the estimation of local commute between these urban areas using the radiation model^8^.

In March and early April 2009, a novel swine-origin influenza A (H1N1) virus was determined to have caused severe respiratory illness in several individuals in Mexico and the United States^9^. By June 2009, nearly 30,000 confirmed cases were reported in 74 countries, prompting the World Health Organization (WHO) to officially declare the start of the 2009 influenza pandemic^10^. The first case of pandemic influenza in Rwanda’s neighboring country, Kenya, was identified on June 29, 2009, and by early October, Rwanda had detected its first case as well^11,12^. While historical information on the burden and effect of Influenza-Like Illness (ILI) in African countries is scarce, projects such as the Influenza Sentinel Surveillance (ISS) system^13^ and Strengthening Influenza Sentinel Surveillance in Africa^14^ (SISA), launched in 2008 and 2011 respectively, have improved influenza surveillance and monitoring. The availability of H1N1 outbreak and GIS data for Rwanda^11,15^, as well as the fact that it is a hotspot region for EIDs^16^ enabled us to construct this modeling framework for Rwanda and simulate the spread of pandemic influenza across it. The ISS system in Rwanda allowed for the comparison of modeling results with data on the detection of actual cases for the 2009 pandemic influenza^11^.

Our aims were to build a model that could simulate the spread of diseases regionally at a very fine scale, initiate the infection from anywhere in the region, test the effect of control measures such as vaccination on the outbreaks, and derive inferences about patterns of disease spread. The resulting work provides a framework for modeling the likely spread of infectious diseases in data-scarce regions important for emerging infectious diseases and can thus be valuable for planning and control purposes in real outbreak scenarios.

## Methods

### Creation of geospatial network

**Figure 1:**
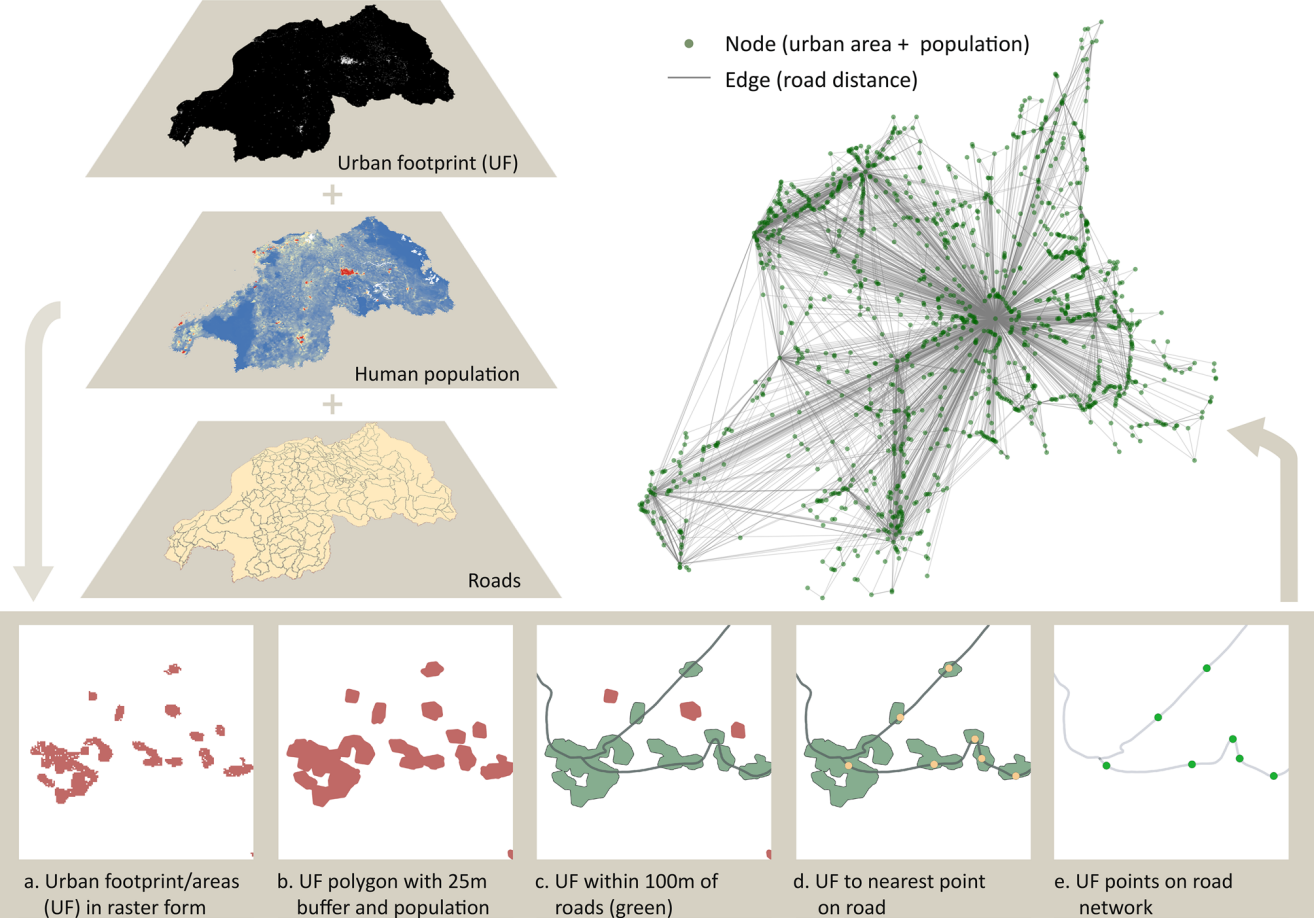
Network creation process depicting how the three GIS layers (urban footprint^7^, population^17,18^, and roads^15^) were combined to form a network. (**a**) Section of urban footprint (UF) layer in raster form. (**b**) Conversion of the UF raster to polygon with added population information and 25 m buffer. (**c**) Subset urban areas that are near roads (100 m including the buffer). (**d**) Conversion of urban areas into points on the road network. (e) Calculation of road distances between urban points. (Edges with less than one human commuting between nodes have not been displayed in the generated network to facilitate network visualization).

We generated the geospatial network by combining three spatial datasets: high resolution human settlement/urban footprint data derived from satellite imagery^7^ (~ 12 m GUF: Global Urban Footprint), human population distribution data by Worldpop^17,18^, and the Rwandan road network data^15^ (Fig. 1). The urban footprint raster layer was converted into polygons, a 25 m buffer was created around them, and the corresponding population for these polygons was added using Worldpop data. All polygons within 75 m (for an effective distance of 100 m) from the roads were selected, and the nearest point from their centroids to the roads was determined. Thereafter, the shortest road distances between all points were calculated, resulting in a network whose nodes corresponded to urban settlement points with their associated populations and edges to the shortest road distances between all points. The network was pruned to keep only those nodes with populations of 10 or more people to improve computation speed for subsequent analyses. The geospatial network was constructed in ArcGIS 10.4 software by ESRI (2016, Redlands, CA), using its Network Analyst tool.

### Human mobility

In order to assist in predicting disease propagation, the edges of the network were weighted by commuting rates: that is, the proportion of the population in a source node that commutes to a destination node. In the absence of readily available commuting data for Rwanda, we chose to estimate these commuting rates by adapting the radiation model described by Simini et al.^8^ to incorporating road distances instead of straight-line Euclidean distances. We also used the same commuting proportion as Simini et al.^8^, 11%. The expected commuting rate $\sigma_{ji}$ from a source node $j$ and a destination node $i$ is:

1 $$E\left[ {\sigma_{ji} } \right] = \left\langle {\sigma_{ji} } \right\rangle = \sigma_{j} \frac{{n_{j} n_{i} }}{{\left( {n_{j} + s_{ji} } \right)\left( {n_{j} + n_{i} + s_{ji} } \right)}}$$

where $n_{j}$ and $n_{i}$ are the populations for nodes $j$ and $i$, and $s_{ji}$ is the total population (excluding $j$ and $i$) of locations within the same road distance to $j$ as that between $j$ and $i$. The total commuting rate of individuals in $j$ is $\sigma_{j} = N_{c} /N$ = 11%, where $N_{c}$ is the total number of commuters and $N$ is the total population in the country.

### Disease model

**Figure 2:**
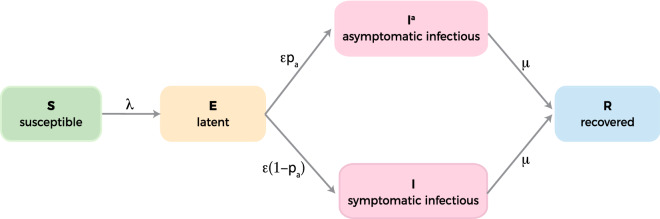
Flow diagram of the SEIR transmission model (boxes represent an SEIR compartment state and arrows represent the rate of transition from one state to another). A susceptible individual in each subpopulation (network node) contracts the infection at rate $\lambda$ and enters the latent compartment. The individual is infected but not infectious during the latent period (${\epsilon }^{-1}$ days). Latent individuals subsequently transition to being either asymptomatic infectious or symptomatic infectious with rates $\epsilon {p}_{a}$ and $\epsilon (1-{p}_{a})$ respectively. Infectious individuals recover permanently at rate $\mu$.

To test the created network as a framework upon which to model infectious diseases, we simulated the spread of pandemic influenza A H1N1 (pH1N1) via a discrete stochastic SEIR compartmental model, in which the disease spread is simulated both within and between nodes (i.e. urban/built-up areas with their associated populations). Using the metapopulation model approach, each node in the network underwent the compartmental model process that simulated disease progression in its population as a result of both within-node and between-node outbreak spread. In the SEIR model, a susceptible person becomes infected upon contact with an infectious individual and transitions to the latent compartment, after which the individual becomes infectious (symptomatic or asymptomatic) and subsequently recovers with immunity to future infections (Fig. 2). All disease parameters used in the model were obtained from existing literature on influenza and pH1N1 (Table 1) and no additional estimates were made. Transitions across the compartments were sampled from multinomial distributions with associated probabilities (transition rates) and sizes (number of individuals in the compartment).

**Table 1:** Disease parameter values used in the modeling process.

Variable	Meaning	Value	Source
$R_{o}$	Reproductive rate	1.44	Pourbohloul et al. 2009^32^
$\epsilon^{ - 1}$	Mean latent period (days)	2.62	Tuite et al. 2010^33^
$\mu^{ - 1}$	Mean infectious period (days)	3.38	Tuite et al. 2010^33^
$p_{a}$	Probability of asymptomatic infection	1/3	Longini et al. 2005^25^
$r_{\beta }$	Reduced infectiousness by asymptomatic infection	50%	Longini et al. 2005^25^
$\beta$	Transmission rate	$R_{o} \mu /\left( {r_{\beta } p_{a} + \left( {1 - p_{a} } \right)} \right)$	Balcan et al. 2010^6^
$\lambda$	Force of infection	Equations 2, 3, and 4	Balcan et al. 2010^6^

The force of infection, $\lambda$, is the net infection rate per susceptible in a metapopulation model. Using the method elucidated by Balcan et al.^6^, the force of infection experienced by a susceptible individual in a subpopulation $j$, $\lambda_{j}$, is a function of the force of infection due to local infectious individuals in $j$ ($\lambda_{jj}$) and infections in $j$'s neighbors $i$ ($\lambda_{ji}$) to which individuals from $j$ commute. Incorporating the commuting flows ($\sigma$) and return rates ($\tau$) of the commuting individuals, the formula for $\lambda$ as given by Balcan et al.^6^ is:

2 $$\lambda_{j} = \frac{{\lambda_{jj} }}{{1 + \sigma_{j} /\tau }} + \mathop \sum \limits_{i \in \upsilon \left( j \right)} \frac{{\lambda_{ji} \sigma_{ji} /\tau }}{{1 + \sigma_{j} /\tau }}.$$

The force of infection experienced within node $j$ is due to infectious individuals staying in $j$ and infectious individuals from $j$'s neighbors, $i$, visiting $j$:

3 $$\lambda_{jj} = \frac{\beta}{{N_{j}^{*} }}\left[ {\frac{{I_{j} + r_{\beta } I_{j}^{a} }}{{1 + \sigma_{j} /\tau }} + \mathop \sum \limits_{i \in \upsilon \left( j \right)} \frac{{I_{i} + r_{\beta } I_{i}^{a} }}{{1 + \sigma_{i} }}\sigma_{ij} /\tau } \right].$$

**Figure 3:**
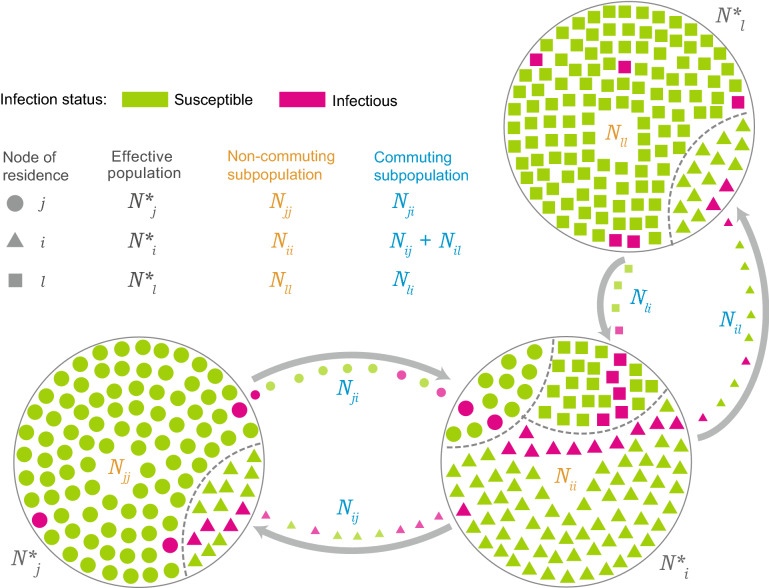
Schematic representation of the commuting flow and how it relates with effective populations and the force of infection. Each node population is divided into subpopulations ${N}_{xy}$, where $x$ represents the node of residence and $y$ represents the node of the actual location at time $t$. Hence, ${N}_{jj}$ refers to the individuals of node $j$ that remain in $j$, ${N}_{ji}$ refers to individuals of node $j$ commuting to node $i$, and ${N}_{j}^{*}$ refers to the effective population of node $j$ taking into account its commuting and non-commuting individuals. Three nodes are shown in the figure – $i$, $j$, and $l$ – to represent the various contributions to the force of infection (Eq. 2).

Similarly, $\lambda_{ji}$, the force of infection due to infections in $j$'s neighbors, $i$, to which individuals from $j$ commute is given by:

4 $$\lambda_{ji} = \frac{\beta }{{N_{i}^{*} }}\left[ {\frac{{I_{i} + r_{\beta } I_{i}^{a} }}{{1 + \sigma_{i} /\tau }} + \mathop \sum \limits_{l \in \upsilon \left( i \right)} \frac{{I_{l} + r_{\beta } I_{l}^{a} }}{{1 + \sigma_{l} }}\sigma_{li} /\tau } \right],$$

where $l$ is the set of neighbors for $i$, whose members commute to $i$. $N_{j}^{*}$ and $N_{i}^{*}$ refer to the effective populations of $j$ and $i$ respectively. The effective populations result from the coupling of human mobility with the node populations^6,19^ as described in Eq. 2 and shown in Fig. 3. Instead of using the exact population of a node, the effective population was determined by taking into account the movement of its individuals to neighboring nodes, the influx of individuals from its neighbors into it, and the return of individuals to their respective subpopulations^6^ (Fig. 3). Assuming a commuting return rate ($\tau$) of 1/3 days^6^, the effective population of node $j$, $N_{j}^{*}$, is given by:

5 $$N_{j}^{*} = \frac{{N_{j} }}{{1 + \sigma_{j} /\tau }} + \mathop \sum \limits_{i \in \upsilon \left( j \right)} \frac{{N_{i} }}{{1 + \sigma_{i} /\tau }}\sigma_{ij} /\tau ,$$

where $N_{j}$ and $N_{i}$ are the populations of node $j$ and its neighbors $i$, respectively; $\sigma_{i}$ and $\sigma_{j}$ are the daily total commuting rates for subpopulations $i$ and $j$, respectively; and $\sigma_{ij}$ is the commuting rate from population $i$ to its neighbor $j$.

We assumed that the populations were homogeneous with no host-specific attributes, such as age and immune status; closed, i.e. there were no births nor deaths in the country's population during the simulation period; homogeneously mixing so all individuals have the same rates of disease-causing contacts; and there were no behavioral changes that could affect disease transmission during the course of the outbreak and its propagation. We also did not account for differences in the mode of transportation (e.g. bus, car, or bicycle) and assumed that people did not make any travel stops during the course of their commute from one area to another.

### Model experimentation, sensitivity analysis, and validation

We initiated outbreaks by converting a susceptible individual to infectious in specific nodes. In the first scenario, we seeded an infectious individual in Kigali, the capital of Rwanda, and the purported starting location of the 2009 pH1N1 outbreak in Rwanda. The results of this scenario were compared to observed data on the pandemic influenza outbreak in Rwanda^11^, specifically the observed outbreak length and order of infection of different populated areas. Assuming these simulations were sufficiently close to reality, we ran further simulations under various scenarios described below and compared their outcomes with each other (Supplementary Table S1).

To compare disease spread under different starting circumstances, we separately seeded another big city, Rubavu, and a smaller town Kibungo, the second and the last places documented to have had pH1N1 infected individuals^11^.

The effect of vaccinating Kigali's population was simulated assuming a vaccine with 80% efficacy^20,21^. For sensitivity analysis, we varied vaccine efficacies from 50 to 90%, and the vaccine coverage of Kigali's population from 20 to 100%. Effectively immunized individuals (product of Kigali's population, proportion vaccinated, and vaccine efficacy) were considered fully immune and transferred to the "Recovered" compartment prior to start of the simulations. The other seeded cities, Rubavu and Kibungo, were also vaccinated similarly, and comparisons were made across different vaccination scenarios: comparing outbreaks originating in Rubavu/Kibungo without any vaccinations, and those with vaccinations in either Kigali, Rubavu/Kibungo, or both Kigali and Rubavu/Kibungo.

Thirty-four sets of simulations were performed, corresponding to each of the scenarios mentioned above; each scenario was run independently 500 times; therefore, we conducted a total of 17,000 simulations across the study. The results are presented using epidemic curves, tables, and maps, including a heat map depicting the distribution of the average number of infectious individuals across Rwanda. The code for setting up the scenarios and running the simulations is presented in https://doi.org/10.5281/zenodo.4014272. All simulations and analyses, except for network creation, were done with the R statistical software^22^; in particular, packages igraph^23^ and dplyr^24^ were used in the simulation code.

**Figure 4:**
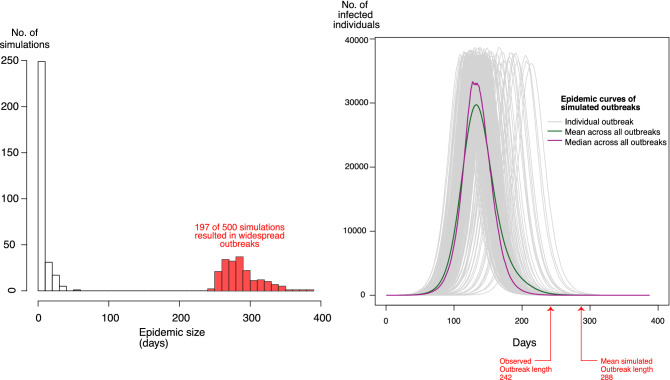
Left: Histogram of simulation lengths (epidemic sizes) of 500 simulations, 197 of which resulted in widespread geospatial outbreaks (infecting greater than 1,000 nodes corresponding to urban areas in Rwanda). Right: Infectious epidemic curves (progression of influenza infections over time) of the 197 simulations that resulted in widespread geospatial outbreaks. The red arrows point out the reported length of the observed 2009 pH1N1 outbreak in Rwanda (242 days) and the mean simulated outbreak length (287.8 days).

**Figure 5:**
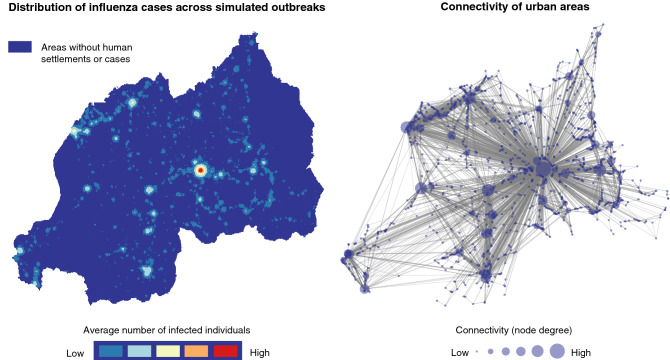
Left: Heat map of the average influenza cases over the course of simulated outbreaks in Rwanda. Right: Map showing connectivity of urban areas in the network.

**Figure 6:**
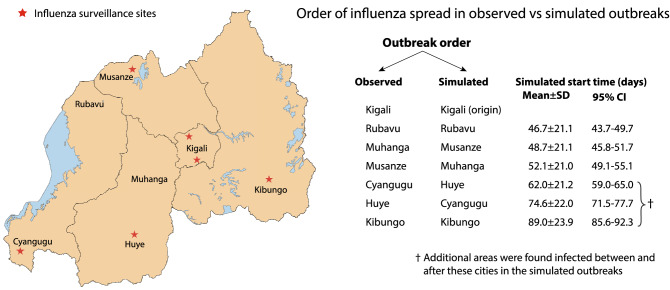
Comparison between observed and simulated outbreak spreads across Rwanda. Left: Map showing the location of influenza surveillance sites^11^ and the cities documented to have confirmed pH1N1 influenza clusters (map made with QGIS 3.6). Right: Comparison between the observed and simulated order of pandemic influenza spread in Rwanda.

**Figure 7:**
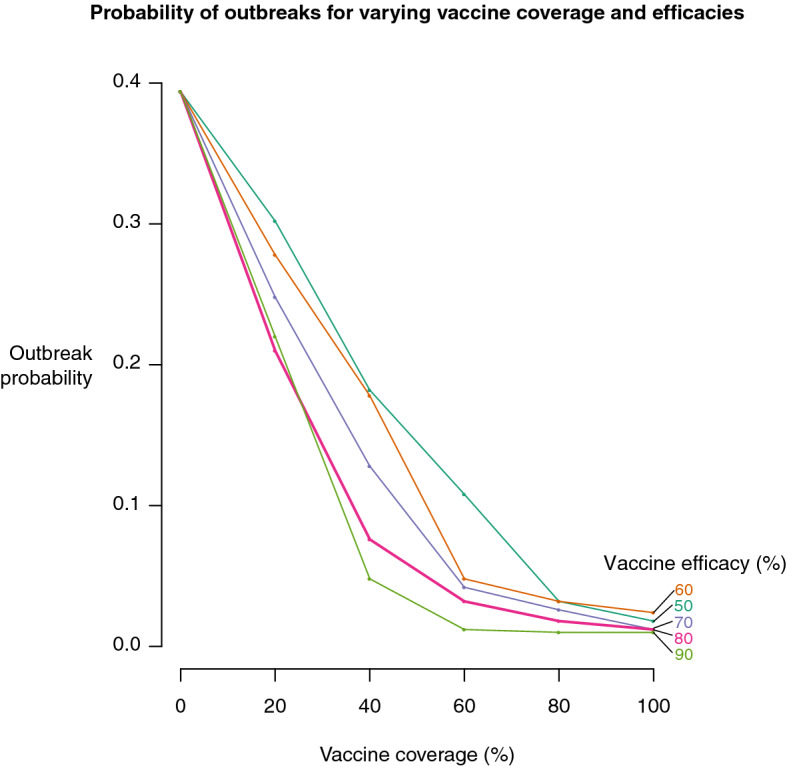
Effect of varying vaccine coverage and efficacy on the probability of an outbreak (the proportion of simulations that resulted in widespread influenza infections across Rwanda).

**Figure 8:**
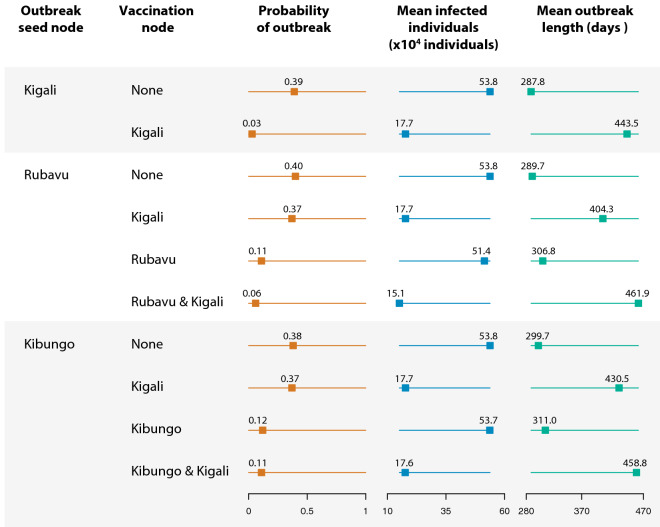
Comparison of the numbers infected and the outbreak lengths for scenarios involving different sites for the start of a potential epidemic and places of vaccinations (60% population coverage with an 80% efficacious vaccine). Outbreak probabilities and numbers of infected individuals were lowest when both Kigali and Rubavu/Kibungo (other cities where outbreaks were initiated) were vaccinated.

## Results

### Epidemic size for infections initiated in Kigali without vaccination

Approximately 39% of the 500 simulations (197/500) in the first scenario resulted in widespread influenza outbreaks, infecting more than 1000 nodes corresponding to urban areas in Rwanda, while 60.6% (303/500) did not spread beyond three nodes and resolved within 52 days. Of the remaining 303 simulations, 97 (32%) did not result in further infections, 190 (63%) did not spread beyond one node and resolved within 52 days, and 16 (5%) spread to a maximum of three nodes and resolved within 35 days. Going forward, because of our focus on the geospatial spread of pH1N1 in Rwanda, we consider those simulations resulting in widespread incidence of influenza in Rwanda as outbreaks. The length of all simulations and infectious curves of the 197 outbreaks are shown in Fig. 4. The mean and median outbreak lengths were 287.8 (IQR: 26.4) and 284.0 (IQR: 33.0) days, respectively.

The distribution of average number of influenza cases across simulated outbreaks is presented in Fig. 5 along with a network map showing the connectivity of urban areas via commuting processes over roads. Areas with greater connectivity are influential during outbreak propagation events and, as observed in Fig. 5, they are also associated with higher number of influenza cases.

### Order of outbreak spread

The laboratory confirmation of new influenza clusters during the 2009–10 influenza outbreak in Rwanda^11^ identified the first cluster in Kigali, followed by Rubavu, Muhanga, Musanze, Cyangugu, Huye, and finally Kibungo (Fig. 6). Under the first simulation scenario, we started the outbreaks in Kigali, and the subsequent order of infection in the aforementioned cities is comparable to the observed outbreak order, with Rubavu, Musanze, and Muhanga being identified as the next three areas to be infected after Kigali (Fig. 6). Further, there were many more areas found to be infected in the simulation results beyond those observed during the actual outbreak, implying that undetected outbreaks in these areas probably occured in reality (Fig. 5).

### Intervention using vaccinations under different scenarios

For outbreaks (simulations resulting in widespread influenza infections) originating in Kigali, vaccinating just 20% of Kigali's population with an 80% efficacious vaccine reduced the outbreak probability by almost half, from 0.39 to 0.21. Increasing vaccination coverage and efficacy resulted in decreasing outbreak probabilities (Fig. 7). The benefits of vaccination beyond 60–80% coverage yielded diminishing gains: for example, increasing the proportion of people vaccinated in Kigali from 60 to 100% only reduced the outbreak probability from 0.018 to 0.012 (Fig. 7).

Simulated outbreaks starting in Rubavu and Kibungo (without vaccination) resulted in similar probabilities of outbreaks and numbers of infectious individuals compared to the one starting in Kigali (Fig. 8). Vaccinating Kigali led to fewer numbers of infections without much effect on the outbreak probabilities. While vaccinating Rubavu and Kibungo resulted in fewer numbers of outbreaks, the numbers of infected individuals remained high. The lowest outbreak probabilities and numbers of infected individuals were observed when both Kigali and the cities where outbreaks were initiated (Rubavu and Kibungo) were vaccinated.

## Discussion

We simulated the spatial spread of an epidemic by creating a fine-scale network of urban areas using satellite-derived imagery and GIS datasets and incorporating human mobility over the resulting network. This is the first time an infectious disease has been modeled utilizing these kinds of georeferenced datasets at such a fine geographic scale. Using freely and publicly available data to create the Rwanda urban area network, running a set of 500 simulations over this network took approximately 11 h to run on a 16 GB RAM machine. The resulting discrete stochastic metapopulation model followed the duration of the observed outbreak (Fig. 4) as well as the order of infection (Fig. 6) of the pandemic influenza A (pH1N1) in Rwanda during 2009–10^11^, illustrating its potential utility in predicting real outbreak scenarios.

Wane et al.^11^ described the clinical and epidemiological features of the pH1N1 outbreak in Rwanda and the public health response by the Rwandan government for its control and mitigation. In 2008, approximately one year before the outbreak, an influenza surveillance system was established in six public hospitals in the capital city of Kigali and the other four provinces of Rwanda (Fig. 6). These sentinel sites served to systematically identify Influenza-Like-Illness and Severe Acute Respiratory Illness cases, and sourced and shipped samples from enrolled cases to the National Reference Laboratory in Kigali. Rwanda's response to the outbreak involved two phases: the containment phase comprising "intensive contact tracing, laboratory testing of suspect cases, mass communication, oseltamivir (Tamiflu) distribution to suspect and laboratory-confirmed cases, and school closure"^11^; and the mitigation phase, initiated upon widespread transmission of pH1N1^11^. The mitigation phase consisted of decentralized outbreak management to the district hospitals, targeted laboratory testing, restricted use of oseltamivir, and enhanced surveillance.

Laboratory testing for pH1N1 in the mitigation phase was limited to 1–5% of suspect cases in an outbreak cluster with a maximum of 10 samples, as well as to severely ill or high-risk suspect cases (e.g. those with asthma or HIV/AIDS) due to cost and logistics. The exact magnitude, path of spread, and distribution of the pH1N1 outbreak in Rwanda are therefore difficult to determine, considering that about 33% of influenza cases can be asymptomatic^25^ and not all suspected influenza cases were tested. Hence, while the actual outbreak was observed to last from early October, 2009 to May 31, 2010, it is likely that the outbreak actually started earlier and finished later than what was observed. Our simulated outbreak length of 287.8 (IQR: 26.4) days encompasses the observed outbreak length of approximately 242 days and possibly provides a more realistic estimate of the actual outbreak duration (Fig. 4).

Pandemic influenza was first confirmed in Kigali, followed by the West, South, and North provinces, and lastly the East province (at the Kibungo sentinel site)^11^. Three foreign nationals arriving from the US and Europe were confirmed positive for pH1N1 as well, and were thought to have acquired the infection outside of Rwanda. Districts with the influenza sentinel sites accounted for the majority of the confirmed pH1N1 cases, showing a possible lack of reporting from districts without surveillance centers. Thus, given the bias of detection around surveillance centers and the fact that only 1–5% of suspect cases in infection clusters were tested, the exact, complete order of the outbreak in Rwanda cannot be accurately quantified. Despite this limitation, our simulations depicted a similar order compared to reports of these sentinel sites (Fig. 6) and identified additional areas which may be of interest for future surveillance and control activities (Fig. 5).

The relative importance of cities and towns during an outbreak depends upon their connectivity with other locations, which in turn is associated with their population sizes and distances by road from each other. Due to the nature of the urban satellite data used to create the network, some cities may have been divided into further subcomponents. For example, Cyangugu appears to have been fragmented into multiple nodes (Supplementary Figure S1), each having lesser populations than that of the city itself. As a result, its overall importance is reduced (via the radiation commuting flow model) and its nodes get infected later than when they would have if they were combined. This division into subcomponents could be addressed by increasing the buffers around urban areas prior to network formation or by further ground-truthing of the urban areas before modeling.

Like any epidemiological modeling study, we made several assumptions that led to a simplification of the modeling process and greatly reduced the computational power needed to run it. We incorporated stochasticity in the model to account for some of the assumptions. In addition to assuming homogeneous populations and mixing within them, we did not account for changes in behavior which could affect disease-causing interactions and therefore alter the course of an outbreak^26,27^. With respect to the network creation process, we incorporated only those urban areas that were within 100 m from the road network in Rwanda, thereby excluding apparently unconnected areas from the network model. Further improvements could be made by creating a more inclusive network via addition of buffers around unconnected areas, joining areas within a reasonable distance from each other, and considering other means of travel or impedances to travel, such as land elevation. Most importantly, we assume that the urban footprint data used to create the network reasonably accounts for the human dwellings important for the outbreak spread. Despite the above assumptions, this model seems to capture well the observed epidemic in Rwanda, as it was comparable with the actual outbreak data from 2009–10 with respect to the epidemic length and spatial spread of cases. The model is also relatively more realistic with respect to human mobility than those using straight-line Euclidean distances between places because it incorporates human movement via roads. Further validation or ground-truthing of this approach to estimating human movements using actual mobility data would be a valuable next step for studies using this modeling process.

Vaccination was instrumental in reducing the probability of an epidemic, with the probability sharply declining with increasing vaccination percentages of up to 60% of Kigali's population. An 80% coverage afforded nearly as effective protection as vaccinating the entire population of Kigali, a highly unrealistic scenario, since complete coverage is difficult to attain. Of note is that when the infections were initiated in Rubavu and Kibungo, slightly lower numbers of outbreaks took place, with approximately the same number of infections as when the outbreaks started in Kigali. This outcome resulted because once the outbreaks reached Kigali, an important node due to its high connectivity with the other areas in the network, they spread from this city to the rest of the network. Vaccinating the population in Kigali while starting the outbreaks from Rubavu and Kibungo reduced the total number of infections, but did not reduce the probability of an outbreak. Vaccinating Rubavu, Kibungo, and Kigali reduced both the probability of outbreaks and numbers of infections, whereas vaccinating Rubavu and Kibungo reduced the probability of outbreaks but did not affect the number of infections.

To reduce the impact of an outbreak in terms of numbers of people who get sick, vaccinating cities with greater connectivity and higher populations is most effective. However, to reduce the probability of an outbreak in the first place, it is important to detect cases early, and to target the source of the outbreak, regardless of its connectivity or population size. This is especially important given the fact that vaccines may not be available at the start of an outbreak^28^. Strengthening public health surveillance to provide early warnings of emerging infections and initiation of control responses at the source is important if we are to protect ourselves from emerging infections^29^. The influenza surveillance system set up in Rwanda^11^ is critical if outbreaks are to be prevented at the source before widespread human epidemics^30^. The rapid identification and restriction of Ebola and Marburg virus diseases in Uganda and the Democratic Republic of Congo demonstrate the returns for investing in surveillance systems and diagnostic capacities^31^.

In conclusion, our high resolution modeling approach can be useful in predicting the spread of, and plan for, future outbreaks in regions where modeling data are scarce or unavailable. This process can be especially helpful during initial phases of outbreaks, before changes in human behavior affect the course of epidemics. Additionally, while we simulated influenza, it is possible to model other infectious diseases, such as SARS-CoV-2 or Ebola, where mobility can lead to rapid and devastating outbreak propagation^31^. In outbreak scenarios, our tool can be valuable for risk-based disease surveillance, emergency planning, and control purposes. It tells us how an epidemic might spread regionally and informs us that controlling an outbreak at its source is more effective than post-outbreak control measures aimed at large influential areas alone.

## Supplementary Information

Supplementary Information 1.

Supplementary Video 1.

## Data Availability

Data used in this study were accessed from the Global Urban Footprint, Worldpop Rwanda 2010 100 × 100 m resolution population data, and Rwanda Transport Development Agency. The compiled network dataset used for modeling is provided at https://doi.org/10.5281/zenodo.4014272.

## Code availability

All R code used for this project is available at https://doi.org/10.5281/zenodo.4014272.
