# Publisher Correction: Fine scale infectious disease modeling using satellite‑derived data

**DOI:** 10.1038/s41598-021-92411-9

**Published:** 2021-06-30

**Authors:** Nistara Randhawa, Hugo Mailhot, Duncan Temple Lang, Beatriz Martínez‑López, Kirsten Gilardi, Jonna A. K. Mazet

**Affiliations:** 1 One Health Institute, School of Veterinary Medicine, University of California, Davis, USA; 2 University of California, Davis, USA; 3 Department of Statistics, University of California, Davis, USA; 4 Center for Animal Disease Modeling and Surveillance, Department of Medicine and Epidemiology, School of Veterinary Medicine, University of California, Davis, USA

Correction to: *Scientific Reports* 10.1038/s41598-021-86124-2, published online 25 March 2021

The original version of this Article contained an error in Figure 4, where the Y-axis label on the right-hand graph was cropped. The original Figure 4 and accompanying legend appear below.

**Figure 4:**
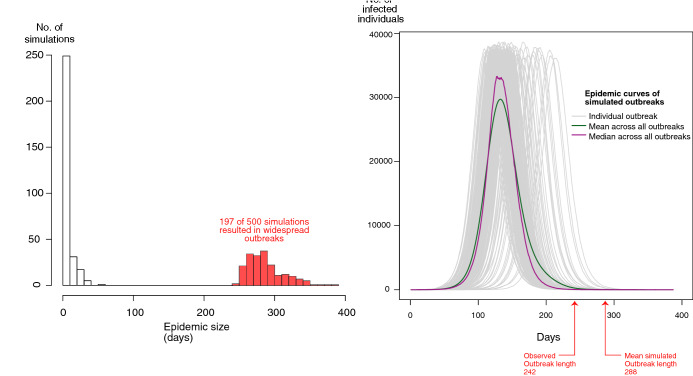
Left: Histogram of simulation lengths (epidemic sizes) of 500 simulations, 197 of which resulted in widespread geospatial outbreaks (infecting greater than 1,000 nodes corresponding to urban areas in Rwanda). Right: Infectious epidemic curves (progression of influenza infections over time) of the 197 simulations that resulted in widespread geospatial outbreaks. The red arrows point out the reported length of the observed 2009 pH1N1 outbreak in Rwanda (242 days) and the mean simulated outbreak length (287.8 days).

The original Article has been corrected.

