# Supplementary material for: Fine scale infectious disease modeling using satellite-derived data
Source: Sci Rep. 2021 Mar 25;11:6946. doi: 10.1038/s41598-021-86124-2 (PMC7994421; doi:10.1038/s41598-021-86124-2)
Supplement: Supplementary file 1 — Supplementary Information 1. [file 41598_2021_86124_MOESM1_ESM.pdf]

Supplementary information

Fine scale infectious disease modeling using satellite-derived data: application to pandemic influenza in Rwanda

Nistara Randhawa[1], Hugo Mailhot[2], Duncan Temple Lang[3], Beatriz Martínez-López[4], Kirsten Gilardi[1], Jonna A.K. Mazet[1*]

[1]One Health Institute, School of Veterinary Medicine, University of California, Davis, USA

[2]University of California, Davis, USA

[3]Department of Statistics, University of California, Davis, USA

[4]Center for Animal Disease Modeling and Surveillance, Department of Medicine & Epidemiology, School of Veterinary Medicine, University of California, Davis, USA

*Corresponding author (jkmazet@ucdavis.edu)

Table S1: Scenarios for the 34 sets of simulations conducted in study

Simulation	Outbreak start	Vaccination locations	Vaccination efficacy	Vaccination coverage	Comparisons made with
1	Kigali	-	-	-	Real outbreak (length and order of spread)
2	"	Kigali	50	20	Simulated outbreaks
3	"	"	60	20	"
4	"	"	70	20	"
5	"	"	80	20	"
6	"	"	90	20	"
7	"	"	50	40	"
8	"	"	60	40	"
9	"	"	70	40	"
10	"	"	80	40	"
11	"	"	90	40	"
12	"	"	50	60	"
13	"	"	60	60	"
14	"	"	70	60	"
15	"	"	80	60	"
16	"	"	90	60	"
17	"	"	50	80	"
18	"	"	60	80	"
19	"	"	70	80	"
20	"	"	80	80	"
21	"	"	90	80	"
22	"	"	50	100	"
23	"	"	60	100	"
24	"	"	70	100	"
25	"	"	80	100	"
26	"	"	90	100	"
27	Rubavu	-	-	-	"
28	"	Kigali	80	60	"
29	"	Rubavu	80	60	"
30	"	Kigali & Rubavu	80	60	"
31	Kibungo	-	-	-	"
32	"	Kigali	80	60	"
33	"	Kibungo	80	60	"
34	"	Kigali & Kibungo	80	60	"

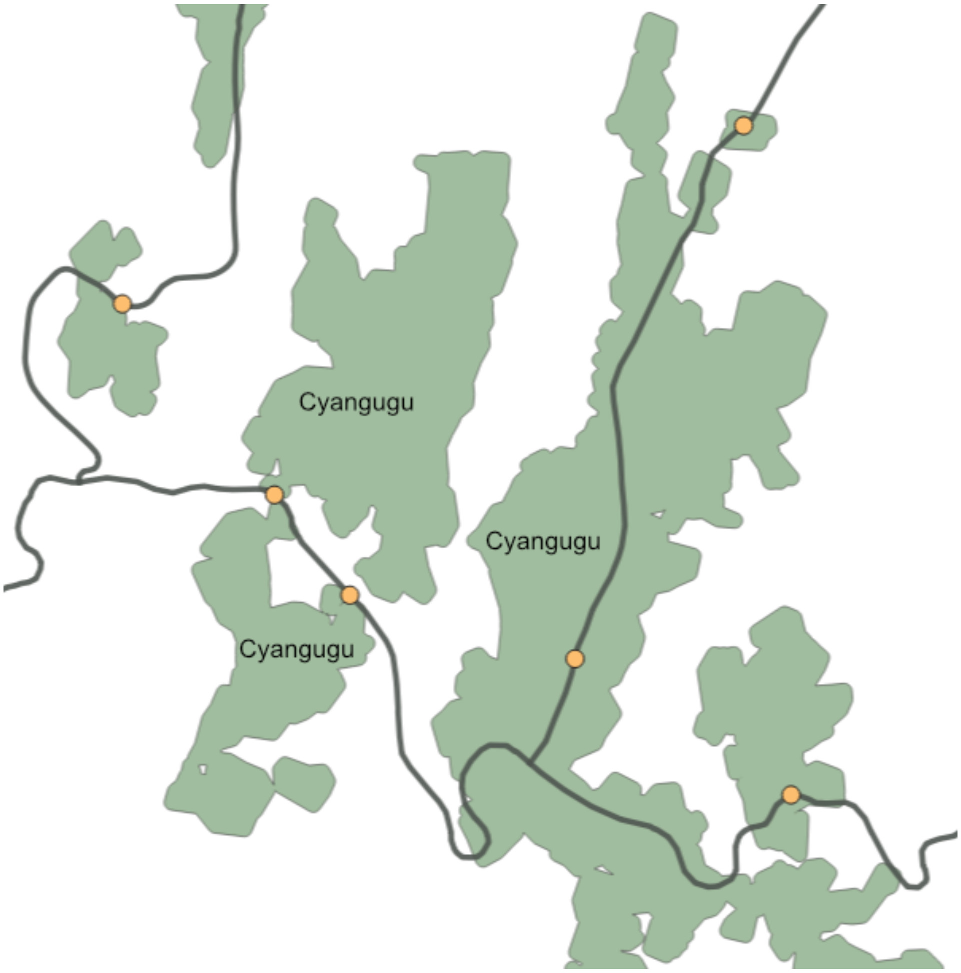

Figure S1: Map showing Cyangugu and the multiple subcomponents of urban built-up areas representing it.
